# Management of anterior cruciate ligament rupture in patients aged 40 years and older

**DOI:** 10.1007/s10195-011-0167-6

**Published:** 2011-11-11

**Authors:** Claudio Legnani, Clara Terzaghi, Enrico Borgo, Alberto Ventura

**Affiliations:** 1 U.O.S.D. Chirurgia Articolare Mininvasiva (Minimally Invasive Articular Surgery Unit), Istituto Ortopedico G. Pini, Milan, Italy; 2 Scuola di Specializzazione in Ortopedia e Traumatologia, Università degli Studi di Milano, Milan, Italy

**Keywords:** Anterior cruciate ligament, ACL reconstruction, Over 40

## Abstract

The aim of anterior cruciate ligament (ACL) reconstruction is essentially to restore functional stability of the knee and to allow patients to return to their desired work and activities. While in the young and active population, surgery is often the best therapeutic option after an ACL tear, ACL reconstruction in middle-aged people is rather more controversial due to concerns about a higher complication rate. The purpose of our article is to establish, through a systematic review of the literature, useful decision-making criteria for the management of anterior cruciate ligament rupture in patients aged 40 years and older, guiding surgeons to the most appropriate therapeutic approach. Various reports have shown excellent results of ACL reconstruction in patients over the age of 40 in terms of subjective satisfaction, return to previous activity level, and reduced complication and failure rates. Some even document excellent outcomes in subjects of 50 years and older. Although there are limited high-level studies, data reported in the literature suggest that ACL reconstruction can be successful in appropriately selected, motivated older patients with symptomatic knee instability who want to return to participating in highly demanding sport and recreational activities. Deciding factors are based on occupation, sex, activity level of the subject, amount of time spent performing such highly demanding activities, and presence of associated knee lesions. Physiological age and activity level are more important than chronological age as deciding factors when considering ACL reconstruction.

## Introduction

The anterior cruciate ligament (ACL) rupture is one of the most common knee injuries in sports.

In the young, athletic patient, surgical treatment of an ACL tear is commonly performed to restore knee kinematics, reducing the risks of subsequent injury and the progression of degenerative changes. As average age and life expectancy are rising, physical activity levels in the elderly population are increasing, and ACL injuries are becoming more frequent in the over-40 population.

Conservative treatment has traditionally been reserved for patients who do not perform highly demanding activities, and consists of modifying activities, quadriceps muscle strengthening, proprioception exercises, and bracing [1–3]. Ciccotti et al. [1] observed a satisfactory outcome in 83% of conservatively treated ACL-deficient knees in a population ranging in age from 40 to 60 years, despite a re-injury rate of 37% and remarkable modifications of activity levels and lifestyles.

Recent studies underline that conservative treatment provides inadequate results, as patients have to cope with instability problems as they return to sport or leisure activities, with increased risk of residual instability and chronic associated injuries [4, 5].

Several surgical strategies exist for the treatment of an ACL lesion. The patellar tendon [6], the iliotibial tract [7], and the hamstring tendons [8] are widely used for intra-articular reconstruction. However, autologous grafts have some well-recognized drawbacks related to donor site morbidity and delayed return to pre-injury levels [9, 10]. Allograft tendons have reportedly produced excellent clinical outcomes, but these grafts bring the risk of infection and disease transmission, and sterilization could cause a weakening of the tissue [36]. For these reasons, their use has been confined to ACL revision surgery [11, 12], although there are studies that recommend allograft ACL reconstruction in middle-aged patients [13–15]. Artificial ligaments have been demonstrated to be unreliable in the long term, as synthetic grafts have been shown to be able to induce osteoarthritis in the knee joint instead of preventing it [16, 17].

Currently, ACL reconstruction is becoming more common in active patients over 40 years, and age does not represent the major criteria in the decision-making process for the treatment of the ACL-deficient knee in the elderly population.

The purpose of our article is to establish, through a systematic review of the literature, useful decision-making criteria that will guide surgeons to the most appropriate therapeutic approach for the ACL-deficient knee in middle-aged patients.

## Materials and methods

### Types of studies

Studies in English pertaining to all levels of evidence and reporting on subjects aged 40 years and older with symptomatic ACL ruptures undergoing surgical reconstruction were considered. No date limits were set. Comparison groups were included (either control or alternative surgical intervention). Case reports, review articles, abstracts, and expert opinions or editorial pieces were excluded.

### Search strategy

Searches were carried out using the following databases: Pubmed/MEDLINE, CINAHL, SCOPUS, Embase, and Ovid. The following keywords were used: “over 40” OR “middle aged” OR “elderly” AND “knee” AND “anterior cruciate ligament” AND “reconstruction.” The abstracts of all hits were reviewed. Duplicates were sifted out and references were hand screened for relevant citations.

### Data extraction

Study characteristics such as year of publication, study population, mean age, level of evidence, graft choice, type of surgical technique, and follow-up duration were extracted and collected by two reviewers, and checked by a third. An electronic database was created. The Oxford Centre for Evidence Based Medicine (CEBM) hierarchy of evidence was used to determine the level of evidence of studies [18]. Outcomes of interest included subjective assessment, knee stability measurements, changes in activity level, and complication rates.

## Results

### Search results

Our search retrieved 732 articles. Overall, 17 studies met all the inclusion criteria for this review (Table 1). All were published between 1996 and 2011.

**Table 1:** Summary of studies included in the present paper reporting results of ACL reconstruction in middle-aged patients

Author	No. of patients	Level of evidence	Mean age (range)	Follow-up period (range)	Graft type
Barber et al. [19]	33	II prospective clinical trial	44 years (40–52)	21 months (12–36)	12 BPTB, 21 Allograft
Heier et al. [24]	45	III retrospective cohort study	44.6 years (40–62)	37 months (24–96)	BPTB
Plancher et al. [25]	75	III retrospective cohort study	45 years (40–60)	55 months (26–117)	BPTB
Viola et al. [20]	11	III retrospective case–control study	42.6 years (40–47)	29 months (12–42)	BPTB
Brandsson et al. [21]	30	III retrospective case–control study	43 years (40–51)	31 months (22–60)	BPTB
Zysk et al. [22]	102	III retrospective case–control study	46 years (40–59)	29 months (12–46)	Primary suture with or without semitendinosus tendon augmentation
Kuechle et al. [13]	47	III retrospective cohort study	45 years (40.2–60.8)	59.7 months (24–110)	Allograft
Barrett et al. [26]	63	III retrospective cohort study	47.13 years (40–58) Allograft	36.4 months (24–74) Allograft	38 Allograft, 25 BPTB
			44.52 years (40–54) BPTB	44.4 months (24–99) BPTB	
Javernick et al. [27]	84	III retrospective cohort study	45 years (40-56)	43 months (12–72)	Hamstrings
Marquass et al. [29]	28	IV case series	45.4 years (40–61)	30.4 months (14–57)	Hamstrings
Khan et al. [30]	21	IV case series	44 years (40–56)	24.5 months (12–37)	Hamstrings
Barber et al. [15]	11	III retrospective case–control study	44 years (40–56)	35 months (24–58)	BPTB allograft
Blyth et al. [28]	31	III retrospective cohort study	54.5 years (50–66)	46 months (24–95)	10 BPTB, 21 hamstrings
Stein et al. [14]	19	IV case series	54 years (49–64)	24 months (9–48)	Allograft
Dahm et al. [31]	35	IV case series	57 years (50–66)	72 months (25–173)	23 Allograft, 12 BPTB
Trojani et al. [32]	18	IV case series	57 years (51–66)	31 months (12–59)	Hamstrings
Osti et al. [23]	20	III retrospective case–control study	56 years (50–62)	32 months (24–49)	N/a

*N/a* not available, *BPTB* bone-patellar tendon-bone

The search resulted in only one level II prospective randomized control trial [19]. Mostly level III studies were reported, either case–control [15, 20–23] or retrospective cohort [13, 24–28] studies. Five were case series (level IV) [14, 29–32]. Follow-up periods ranged from 9 months to 14 years.

### Clinical assessment

A remarkable improvement in Lysholm scores was noted in most studies (Table 2), with results ranging from 88.5 to 95. Only one study, Zysk et al. [22] reported abnormal and severely abnormal (97% PT vs. 85% HT) results, but these results were biased due to the different patient populations (acute ACL injury only) and reconstruction techniques (primay suture with or without semitendinosus augmentation).

According to the IKDC evaluation form, 64–93% patients achieved good or excellent postoperative scores. Lower functional outcomes were observed in patients who had significant underlying osteoarthritis and in those with concomitant cartilage lesions [14, 28].

Ten studies [15, 19–21, 26–31] used the Tegner activity score to evaluate the levels of activity. In all cases, ACL reconstruction produced average or satisfactory results, with scores ranging from 4.1 to 6.6.

These results were consistent throughout the studies, suggesting that the majority of patients returned to their pre-injury activity levels. However, most patients in the over-40 age group are not involved in—and therefore do not return to–high-level sporting activities (sports involving pivoting, cutting, and jumping).

**Table 2:** Results of subjective and objective evaluations

Author	IKDC score	Lysholm score	Tegner score	Arthrometer (laxity ≤ 3 mm vs. normal knee)
Barber et al. [19]	N/a	95	5.7	15 (79%)
Heier et al. [24]	A: 4 B: 25 C: 14 D: 2	91	N/a	31 (78%)
Plancher et al. [25]	A: 21 B: 49 C: 5 D: 0	94	N/a	50 (67%)
Viola et al. [20]	A: 1 B: 8 C: 2 D: 0	88.5	5.3	7 (64%)
Brandsson et al. [21]	A: 10 B: 12 C: 6 D: 2	91	5	21 (70%)
Zysk et al. [22]	N/a	88 Augmentation 80 Primary suture	N/a	23 (66%) Primary suture 60 (90%) Augmentation
Kuechle et al. [13]	N/a	89.7	N/a	22 (81%)
Barrett et al. [26]	N/a	91 Allograft 92 BPTB	4.1 Allograft 4.3 BPTB	33 (86%) Allograft 24 (96%) BPTB
Javernick et al. [27]	N/a	94	5	N/a
Marquass et al. [29]	83.4	91.5	4.5	16 (57%)
Khan et al. [30]	83	92	6	19 (90%)
Barber et al. [15]	N/a	88.8	6.6	10 (91%)
Blyth et al. [28]	A: 5 B: 20 C: 6 D: 0	93	5.2	11 (41%)
Stein et al. [14]	N/a	92	N/a	18 (95%)
Dahm et al. [31]	90	92	4.3	N/a
Trojani et al. [32]	A: 7 B: 7 C: 3 D: 1	N/a	N/a	N/a
Osti et al. [23]	91	89	N/a	15 (75%)

*N/a* not available, *IKDC* International Knee Documentation Committee, *BPTB* bone-patellar tendon-bone

### Anteroposterior laxity

The KT-1000 arthrometer (MEDmetric Corp., San Diego, CA, USA) or the Rolimeter (Aircast, Summit, NJ, USA) were used in most of the studies considered to instrumentally assess the amount of anteroposterior dislocation. In one paper, the Telos stress system (Metax GmbH, Marburg, Germany) was used [32]. Two studies reported the results of Lachman and pivot-shift tests [27, 31]. All reports showed an improvement in mean residual differential laxity. In seven studies, >75% of patients had a side-to-side difference of less than or equal to 3 mm [13–15, 19, 24, 26, 30].

### Comparison with control group

Four retrospective case–control studies compared the outcomes of ACL reconstruction in subjects aged 40 years and older with those for a group of younger patients [15, 19, 21, 23].

Barber et al. [19] did not report any statistically significant difference between a group with an average age of 27 years (91% excellent or good results) and a group with an average age of 44 (89% satisfactory outcomes). Brandsson et al. [21] compared the results of ACL reconstruction in patients aged less than 24 years and over 40 years: no statistically significant differences were found in terms of IKDC, Lysholm knee score, and Tegner activity level. There were significantly more patients with subjectively excellent results in the elderly group.

### Graft choice

Seven studies used ipsilateral bone-patellar tendon-bone (BPTB) autografts for ACL reconstruction [19–21, 24–26, 31]; five papers reporting autologous hamstring reconstruction provided similar results [27, 29, 30, 32]. One study used a mixed population of BPTB and hamstring ACL reconstructions without discriminating between the outcomes of the two different reconstruction techniques [28].

Six papers reported the results of allograft ACL reconstruction in middle-aged patients using freeze-dried fascia lata, BPTB, or Achilles allograft [13–15, 19, 26, 31]. No evidence of disease transmission or tissue rejection was noted.

Barrett et al. [26] compared ipsilateral bone-patellar tendon-bone (BPTB) autologous graft with BPTB allograft in a population of 63 patients aged >40 years undergoing ACL reconstruction. Dahm et al. [31] reviewed the records of 34 patients aged 50 years or over after ACL reconstruction in 35 knees with BPTB allograft or autograft. Both authors reported that there were no statistically significant differences in the outcomes between patients treated with autografts or allografts.

### Complication rates and graft failures

Overall, the following major complications were reported: eight deep vein thromboses and one lung embolism. The intolerance to hardware rate ranged from 0 to 28%. All symptomatic cases resolved after removal of the painful fixation device. Wound complications, which included either superficial wound infections or wound healing problems, ranged from 0 to 6%. One study reported complications arising from sterile synovitis [26]. Loss of postoperative motion was the most recurrent complication among the studies considered, and ranged from 0 to 27%. The highest graft failure rate (9%) was reported by Dahm et al. [31] (Table 3).

**Table 3:** Complications and failure rates

Author	Complications	Graft failures (%)
Barber et al. [19]	1 (3%) loss of postoperative motion	0 (0)
Heier et al. [24]	1 (2%) loss of postoperative motion 1 (2%) anterior knee pain	2 (4)
Plancher et al. [25]	4 (4%) hardware intolerances 1 (1%) patellar ligament inflammation 3 (3%) losses of postoperative motion	0 (0)
Viola et al. [20]	1 (9%) loss of postoperative motion	0 (0)
Brandsson et al. [21]	2 (6%) bleeding complications 8 (27%) losses of postoperative motion	0 (0)
Zysk et al. [22]	1 (1%) bleeding complication 6 (6%) losses of postoperative motion 7 (6.9%) deep vein thromboses 1 (1%) lung embolism	0 (0)
Kuechle et al. [13]	2 (4%) superficial wound infections 13 (28%) hardware intolerances 2 (4%) losses of postoperative motion	1 (2)
Barrett et al. [26]	1 (2%) anterior knee pain 1 (2%) sterile synovitis	1 (2)
Javernick et al. [27]	0 (0%)	0 (0)
Marquass et al. [29]	None reported	0 (0)
Khan et al. [30]	1 (5%) superficial wound infection 1 (5%) deep vein thrombosis	0 (0)
Barber et al. [15]	None reported	0 (0)
Blyth et al. [28]	2 (6%) wound healing problems	0 (0)
Stein et al. [14]	2 (8%) recurrent knee effusions	0 (0)
Dahm et al. [31]	2 (16%) hardware intolerances	3 (9)
Trojani et al. [32]	3 (17%) losses of postoperative motion 1 (5%) posterior knee pain 4 (22%) cases of tibiofemoral pain	0 (0)
Osti et al. [23]	None reported	1 (5)

## Discussion

ACL reconstruction in the over-40 population is still an issue of debate, as there is no consensus among surgeons on whether to treat middle-aged patients with an ACL lesion conservatively or surgically.

Conservative treatment was frequently advocated in the past for middle-aged people with an ACL tear. In fact, some orthopedic surgeons worried that ACL reconstruction on older patients could lead to complications such as stiffness, arthrofibrosis, infections, wound healing problems, or thromboembolic disease, and there were concerns that underlying degenerative knee osteoarthritis could prevent a satisfactory outcome [33, 34].

In the study by Ciccotti et al. [1], nonoperative treatment led to satisfactory outcomes in 83% of patients aged 40–60 years with an ACL tear. However, patients had to renounce any return to competitive pivoting sports, and had to cope with knee instability. Limitations in activity levels have also been observed in younger patients. Fitzgerald et al. [4] reported that in 93 patients (aged 15–57 years old) with ACL-related knee instability, only 39 (42%) met their criteria for conservative treatment, and only 22 (24%) succeeded in returning to pre-injury levels after rehabilitation protocol. Recently, Strehl and Eggly [5] reported the results for 37 patients with an ACL tear treated conservatively (age range 16–55). Twelve patients (32.4%) reported good to excellent outcomes and returned to previous sport activities; 25 patients (67.6%) underwent further surgical ACL reconstruction after an average time of 9.3 months after injury.

Using expected-value decision analysis, Seng et al. [35] determined that operative intervention was the optimal treatment strategy in patients aged 40 years or older with an ACL tear. They found that this population was reluctant to accept a risk of possible re-injury, instability, or modified return to activity.

This systematic review aimed to evaluate the outcomes of ACL reconstruction in middle-aged patients in terms of subjective outcomes, knee stability, return to pre-injury function, implant choice, complication rate, and graft failure.

The purpose of our article is to establish, through a systematic review of the literature, useful decision-making criteria that will guide surgeons to the most appropriate therapeutic approach for the ACL-deficient knee in middle-aged patients.

Several studies have demonstrated that, in a middle-aged population with an ACL tear, selected and motivated patients may experience considerable recoveries of function and stability after surgical reconstruction, with a more predictable return to cutting and pivoting sports.

Operative treatment documented favorable outcomes in this patient population with regard to knee stability and patient satisfaction, with results similar to those observed in a younger patient population [15, 19, 21, 23]. Among the four studies reporting on the difference in the outcomes between elderly and young patients, no increased risk of complication (stiffness, arthrofibrosis, infections) was noted in the middle-aged patients compared to the control group [15, 19, 21, 23].

Selection criteria are needed to determine the risk–benefit relationship of nonoperative versus operative management. Key symptoms leading to surgery are considered recurrent giving-way episodes during daily activities, which affect the quality of life of the subject. Objective clinical parameters to assess are the presence of a soft end-point Lachman sign and combined positivity in the pivot-shift test. It has been proven that the existence of significant underlying osteoarthritis and concomitant cartilage lesions (more common among middle-aged patients) can affect the outcomes of ACL reconstruction [33, 34]. This finding has been noticed in some of the studies considered [14, 28].

Data reported in the literature suggest that ACL reconstruction can be successful in appropriately selected, motivated older patients. In order to maximize the outcome, selection criteria must be strict, and the injured knee must not have more than minimal arthritic changes. For this reason, magnetic resonance imaging (MRI) can be useful to screen for concomitant multiple ligament injury, meniscal lesions, or combined cartilage defects. Imaging results could also be useful during surgical planning, driving the surgeon towards the correct graft choice.

Controversy exists regarding the ACL graft choice for the elderly population; although the BPTB autograft has been widely used [19–21, 24–26, 31], hamstring reconstruction has recently gained in popularity because of reduced donor site morbidity and anterior knee pain [27–30, 32]. Furthermore, patellar tendon harvest could potentially affect the extensor mechanism in the eventuality of joint replacement surgery [27]. Studies advocate that a hamstring autograft could be more appropriate because of the presence in the older patient of patellofemoral chondrosis, patellar tendon weakness, and osteopenia leading to patellar fractures. However, in the only paper reporting on both autologous BPTB and hamstring tendon graft ACL reconstruction, no data on the differences between the outcomes of the two different surgical techniques were provided by the authors [28]. Various authors prefer allografts [13–15] in order to reduce donor site morbidity and shorten operative and rehabilitation times. We found only two studies that provided a subgroup analysis comparing outcomes in autograft and allograft ACL reconstructions [26, 31]; no statistically significant difference in the outcomes between the two groups was reported.

Based on the data present in the literature and on our personal experience, we developed a decision-making strategy for the treatment of the ACL-deficient knee in the elderly population. Treatment of ACL injuries should fit individual patient needs. Deciding factors when determining the appropriate therapeutic decision are based on occupation, sex, activity level of the subject, amount of time spent in highly demanding activities, and presence of associated knee lesions. Physiological age and activity level are more important than chronological age as deciding factors when considering ACL reconstruction.

We commonly propose surgical treatment in symptomatic patients who express the need to restore their pre-injury activity levels, regardless of their age. Clinical parameters leading to surgery are considered positivity to knee laxity tests and recurrent giving-way episodes during daily activities, which lower the quality of life of the subject. In our opinion, the restoration of knee kinematics through ACL reconstruction could allow the subject to return to their previous activity level with less risk of further knee damage and the onset of osteoarthritis.

In contrast, we propose nonoperative treatment for patients who do not perform highly demanding activities, who can cope with instability problems, and for whom quality of life is not affected by knee problems. Older patients are more likely to modify their activity levels and try to avoid the practice that caused the injury. For this reason, the indication must take individual factors, such as the level of activity or a subjective feeling of instability, into account. In addition, we exclude patients with systemic diseases or advanced osteoarthritis from surgery.

The main limit of this systematic review is that there was a considerable lack of high-level studies supporting ACL reconstruction in the middle-aged population. This growing body of papers has broadly changed the approach of surgeons towards the management of the ACL-deficient knee in elderly patients. Recently, reports of ACL reconstruction in patients over 50 years have been published [14, 23, 28, 31, 32]. With increasing numbers of activity-related injuries, and to comply with patient requests to return to pre-injury levels, the cutoff age for surgical treatment has been increased. However, at present, there is a limited evidence base for ACL reconstruction in middle-aged patients, so the expertise of physicians still represents the most useful tool in clinical practice. Further randomized trials and comparative studies are required in order to aid surgeons in determining the correct therapeutic approach for the ACL-deficient knee in the elderly population.
